# Early renal impairment is associated with in‐hospital death of patients with COVID‐19

**DOI:** 10.1111/crj.13496

**Published:** 2022-05-19

**Authors:** Pei‐ling Bao, Ke‐lan Deng, An‐long Yuan, Yi‐min Yan, Ai‐qiao Feng, Tao Li, Xiao‐an Liu

**Affiliations:** ^1^ Department of Nephrology, Xiaogan Hospital Affiliated to Wuhan University of Science and Technology The Central Hospital of Xiaogan Xiaogan China; ^2^ Department of Respiratory disease, Xiaogan Hospital Affiliated to Wuhan University of Science and Technology The Central Hospital of Xiaogan Xiaogan China; ^3^ Department of Gastroenterology, Xiaogan Hospital Affiliated to Wuhan University of Science and Technology The Central Hospital of Xiaogan Xiaogan China; ^4^ Xiaogan Hospital Affiliated to Wuhan University of Science and Technology The Central Hospital of Xiaogan Xiaogan China; ^5^ Department of Rehabilitation Medicine, Xiaogan Hospital Affiliated to Wuhan University of Science and Technology The Central Hospital of Xiaogan Xiaogan China

**Keywords:** COVID‐19, early renal impairment, eGFR, mortality, pneumonia

## Abstract

**Introduction:**

Renal impairment is a common complication in coronavirus disease 2019 (COVID‐19), although its prognostic significance remains unknown.

**Objectives:**

This study determines the impact of early renal impairment on the clinical outcome of COVID‐19.

**Methods:**

Patients diagnosed with COVID‐19 and hospitalized in Xiaogan Central Hospital from 20 January to 29 February 2020 were retrospectively included and grouped into two cohorts (cohort with normal renal function and cohort with renal insufficiency) based on the renal function detected on admission. Records of clinical manifestation, laboratory findings and clinical outcome were collected and compared between these two cohorts.

**Results:**

A total 543 COVID‐19 patients were included. Among these patients, 70 patients developed early renal impairment, with an incidence of 12.89%. A significantly higher white blood cell (WBC) count, C‐reactive protein (CRP), erythrocyte sedimentation rate (ESR), serum creatine (Cr), blood urine nitrogen (BUN) and brain natriuretic peptide (BNP) and a significantly lower blood platelet (PLT), lymphocyte count, prealbumin and albumin (ALB) were detected in the cohort with renal insufficiency (*P* < 0.05). Patients with early renal impairment were also associated with higher incidences of haematuria/proteinuria, higher incidences of mortality and prolonged hospitalization duration. The independent risk factors for in‐hospital death included age >65 years old, complication of diabetes, renal impairment on admission (Cr > 73 μmol/L and eGFR < 60 ml/min 1.73 m^2^), WBC > 9.5 × 10^9^/L and ALB < 35 g/L.

**Conclusion:**

Early renal impairment is associated with higher risk of in‐hospital death for patients with COVID‐19. Risk stratification according to renal function can better guide the clinical management of COVID‐19.

**List of abbreviations**
ACE2: angiotensin‐converting enzyme 2
AKI: acute kidney injury
ALB: prealbumin and albumin
ARDS: acute respiratory distress syndrome
BNP: brain natriuretic peptide
BUN: blood urine nitrogen
CK‐MB: creatine kinase myocardial band
COVID‐19: clinical outcome of coronavirus disease 2019
Cr: serum creatine
CRP: C‐reactive protein
CT: computed tomography
eGFR: glomerular filtration rate
ESR: erythrocyte sedimentation rate
Hb: haemoglobin
PCT: procalcitonin
PLT: significantly lower blood platelet
SD: standard deviation
WBC: white blood cells

## 1 INTRODUCTION

Since its outbreak in December 2019 in Wuhan, China, coronavirus disease 2019 (COVID‐19) has widely spread all over the word and become a pandemic that poses a great threat to the health of all human beings.^1^, ^2^ A high mortality rate was observed for COVID‐19, whereas no specific therapeutic strategy has been proven to be effective for its treatment to date.^3^, ^4^, ^5^, ^6^, ^7^, ^8^, ^9^ A better understanding of the risk factors associated with fatality in COVID‐19 can better guide intervention strategies and improve clinical outcome.

Caused by the newly identified coronavirus, severe acute respiratory syndrome coronavirus 2 (SARS‐CoV‐2), which dominantly target pulmonary tissues, COVID‐19 mainly manifests as pneumonia. In severe cases, acute respiratory distress syndrome (ARDS) can develop.^10^ Kidney involvement also commonly occurred. Multiple studies have found that haematuria, proteinuria and, in severe cases, acute kidney injure (AKI) can develop in COVID‐19 patients.^11^, ^12^ The development of AKI during hospitalization has been associated with the high mortality of COVID‐19.^11^, ^12^ However, the prognostic impact of early renal impairment that occurs when first admitted has been rarely reported.

More than 3000 cases of COVID‐19 have been detected in Xiaogan City, Hubei Province, making this one of the most severe city havocked by COVID‐19 in China, ranking only second to Wuhan. As the biggest hospital in Xiaogan City, Xiaogan Central Hospital plays an essential role in treating COVID‐19 and has hospitalized a large number of COVID‐19 patients. Utilizing the abundant clinical data of COVID‐19 in our hospital, the investigators sought to clarify the clinical features and prognostic impact of early renal impairment in COVID‐19 patients.

## 2 MATERIALS AND METHODS

### 2.1 Patients

A total of 543 the patients diagnosed with COVID‐19 and hospitalized in Xiaogan Central Hospital from 20 January 2020 to 29 February 2020 were included in the present study. All patients were diagnosed according to the diagnostic criteria for COVID‐19 proposed in the Chinese Guidelines for the Diagnosis and Treatment of COVID‐19 (version 7) and were all treatment naïve when diagnosed. The investigators excluded cases that had any of the following conditions: (1) patients who have been treated elsewhere before being transferred to our hospital; (2) patients who acquired the COVID‐19 infection during the hospitalization for other diseases; (3) patients who were <18 years old. All clinical observations ended on 28 March 2020, which was the time the last case of COVID‐19 in Xiaogan City was cured. The present study was approved by the ethics committee of our hospital (Ethics committee approval number: XGLY2020‐04‐11).

### 2.2 Data collection

General information, which included age, gender, height, weight, date of disease onset, date of hospitalization, date of discharge or death, complicated diseases and clinical manifestation, were collected through medical records. Furthermore, the laboratory results, which included white blood cells (WBCs), haemoglobin (Hb), blood platelet (PLT), neutrophil, lymphocytes, eosinophil, prealbumin, albumin (ALB), fibrinogen (Fib), C‐reactive protein (CRP), serum creatine (Cr), blood urine nitrogen (BUN), creatine kinase myocardial band (CK‐MB), brain natriuretic peptide (BNP), erythrocyte sedimentation rate (ESR), procalcitonin (PCT), D‐dimer, urine protein and urine occult blood that was first detected on admission, were also collected. In addition, the imaging data of the computed tomography (CT) scan that was first detected on admission was also collected for all patients. For each patient, the glomerular filtration rate (eGFR) was inferred using the GFR‐EPI_2009Scr_ equation based on the Cr level detected on admission. Based on the eGFR, these patients were grouped into two cohorts: the cohort with renal insufficiency (eGFR < 60 ml/min 1.73 m^2^, *n* = 70) and the cohort with normal renal function (eGFR ≥ 60 ml/min 1.73 m^2^, *n* = 473).

### 2.3 Statistics analysis

The SPSS software package 21.0 for Windows (SPSS Inc., Chicago, IL, USA) was used for the statistical analysis. Normally distributed continuous variables are presented as mean ± standard deviation (SD) and between‐group differences assessed using the independent *t* test. Non‐normally distributed continuous variables are presented in median (25% percentile, 75% percentile), and between‐group differences were assessed using the Mann–Whitney *U* test. Between‐group differences with respect to categorical variables were assessed using the Chi‐squared pair‐wise test. Univariate log rank test was performed to identify risk factors for mortality during hospitalization. For variables with statistical significance in the univariate analysis, a further multivariate survival analysis was performed using the Cox regression model to identify risk factors with an independent impact. A *P* value of <0.05 was considered statistically significant.

## 3 RESULTS

### 3.1 Baseline characteristics of the included cases

A total 543 qualified cases were included in the present study. Among these patients, 291 (53.6%) patients were male, and 252 (46.4%) patients were female. The average age of these patients was 52.72 ± 15.25 years old, in which 118 (21.73%) patients were <40 years old, 305 (56.17%) patients were within 41–60 years old and 120 (22.1%) patients were >65 years old. Different complications existed among the included patients, which included hypertension (*n* = 133, 24.5%), cardiovascular disease (*n* = 18, 3.3%), diabetes mellitus (*n* = 65, 12%), chronic liver disease (*n* = 20, 3.7%), chronic renal disease (*n* = 5, 0.9%) and malignant diseases (*n* = 11, 3.3%). According to the severity of COVID‐19, these patients were classified as mild disease (*n* = 6, 1.1%), general type (*n* = 380, 69.98%) and critical type (*n* = 59, 10.87%). The average time of disease onset before admission was 7 days, and the average hospitalization duration was 20 days. The detailed information is presented in Table 1.

**TABLE 1:** Clinical characteristics of COVID‐19 patients on hospital admission

Characteristics	All patients (*n* = 543)	Clinical subtype		*t* or *χ* ^2^	*P* value
		eGFR ≥ 60 (*n* = 473)	eGFR < 60 (*n* = 70)		
Age (years)—median (IQR)	52 (43–63)	51 (41–61)	64 (54–75)	−6.455	<0.01
Age groups (years)—*n* (%)					
≤40	118 (21.73)	113 (23.89)	5 (7.14)	10.055^a^	0.002
41–65	305 (56.17)	273 (57.72)	32 (45.71)	3.568^a^	0.059
>65	120 (22.1)	87 (18.4)	33 (47.14)	29.275^a^	<0.01
Gender—*n* (%)					
Male	291 (53.6)	235 (49.68)	56 (80)	22.534^a^	<0.01
Female	252 (46.4)	238 (50.32)	14 (20)		
BMI (kg/m^2^)	23.79 ± 3.3	23.72 ± 3.27	24.27 ± 3.57	−1.315	0.189
Coexisting diseases—*n* (%)					
Hypertension	133 (24.5)	102 (21.56)	31 (44.29)	17.021^a^	<0.01
CHD	18 (3.3)	11 (2.3)	7 (10)	11.205^a^	0.001
Diabetes	65 (12)	52 (10.99)	13 (18.57)	3.523^a^	0.048
Liver disease	20 (3.7)	19 (4.02)	1 (1.43)	1.152^a^	0.283
Renal diseases	5 (0.9)	1 (0.21)	4 (5.71)	20.239^a^	<0.01
Malignancies	11 (2.03)	9 (1.9)	2 (2.86)	0.28^a^	0.597
Clinical symptoms—*n* (%)					
Fever	489 (90.1)	424 (89.6)	65 (92.86)	0.704^a^	0.401
Cough	379 (69.8)	327 (69.13)	52 (74.28)	0.768^a^	0.381
Fatigue or myalgia	202 (37.2)	177 (37.42)	25 (35.71)	0.076^a^	0.783
Diarrhoea	28 (5.2)	26 (5.5)	2 (2.86)	0.869^a^	0.351
Clinical outcomes—*n* (%)					
Discharge	514 (94.7)	458 (96.83)	56 (80)	34.159^a^	<0.01
Dead	29 (5.3)	15 (21.43)	14 (20)		
Diseased time	7 (4–11)	7 (4–11)	7 (5–7)	−0.928	0.353
Hospitalized days	20 (15–26)	1 (14–26)	22 (18–32)	−1.938	0.047

Abbreviations: BMI, body mass index; CHD, congenital heart disease; eGFR, glomerular filtration rate; IQR, interquartile range.

^a^ The data are presented in median (IQR), *n* (%), where *n* is the total number of patients with available data; diseased time, interval between illness onset and hospital admission; the *P* values for comparing the four groups are from the Kruskal–Wallis test.

### 3.2 Comparison between the cohort with normal renal function and the cohort with early renal impairment

A total of 70 (12.89%) patients had eGFR < 60 ml/min 1.73m^2^ on admission and were classified as early renal impairment. Compared with patients with normal renal function, patients with early renal impairment were significantly older and had a higher incidence of complications, which included hypertension, cardiovascular disease, diabetes mellitus and renal disease (*P* < 0.05). In addition, the cohort with renal impairment was associated with longer hospitalization duration and higher incidence of mortality during hospitalization (*P* < 0.05). More details are presented in Table 1.

In terms of laboratory findings, patients with early renal impairment had significantly higher WBC, CRP, ESR, BUN, Cr and BNP and lower lymphocytes, PLT, prealbumin and ALB, when compared with the normal group (*P* < 0.05). No significant difference in neutrophil, and eosinophil count, and serum Hb, PCT, Fib, D‐dimer and CK‐MB level were observed between the two cohorts (*P* ≥ 0.05). The detailed information is presented in Table 2.

**TABLE 2:** Laboratory data of patients with COVID‐19 on admission

Laboratory parameters	All patients (*n* = 543)	eGFR > 60 (*n* = 473)	eGFR ≤ 60 (*n* = 70)	*t*	*P* value
WBC count (×10^9^/L)	5.66 ± 3.24	5.58 ± 3.23	6.99 ± 3.02	2.246	0.025
Neutrophil (×10^9^/L)	4.4 ± 5.06	4.24 ± 5.23	5.48 ± 3.62	−1.926	0.055
Lymphocyte (×10^9^/L)	1.1 ± 0.54	1.12 ± 0.55	0.94 ± 0.49	2.51	0.012
Eosinophil (×10^9^/L).	0.04 ± 0.17	0.05 ± 0.18	0.02 ± 0.04	1.149	0.251
Haemoglobin (g/L)	132.22 ± 20.81	131.54 ± 20.69	136.73 ± 21.21	−1.948	0.052
Platelet count (×10^9^/L)	187.02 ± 76.39	191.4 ± 77.8	157.69 ± 58.8	4.271	<0.01
CRP (mg/L)	29.5 ± 41.46	25.54 ± 35.19	56.63 ± 65.03	−3.889	<0.01
ESR (mm/h)	48.31 ± 29.33	47.14 ± 29.36	60.01 ± 31.48	−2.208	0.028
PCT (ng/ml)	0.42 ± 3.07	0.43 ± 3.23	0.26 ± 0.23	0.392	0.695
Fib (g/L)	5.21 ± 6.89	4.94 ± 3.09	4.88 ± 1.45	0.156	0.876
D‐dimer (μg/ml)	1.44 ± 4.65	1.37 ± 4.44	1.26 ± 2.87	0.193	0.847
Albumin (g/L)	37.88 ± 4.23	38.06 ± 3.99	37.07 ± 4.2	1.925	0.055
Prealbumin (g/L)	173.02 ± 77.21	178.07 ± 76.94	141.71 ± 72.82	3.713	<0.01
Urea nitrogen (mmol/L)	4.97 ± 4.26	4.28 ± 3.93	7.73 ± 4.76	−5.768	<0.01
Creatinine (μmol/L)	71.77 ± 33.4	64.55 ± 14.43	121.11 ± 67.59	−6.978	<0.01
CK‐MB (ng/ml)	3.49 ± 8.56	3.36 ± 9.17	4.26 ± 4.29	−0.663	0.508
Pre‐BNP (pg/ml)	623.14 ± 1166	516.72 ± 864	1154.96 ± 2054	−2.116	0.039
Proteinuria (±−3+)	49 of 307	37 of 268	12of 39	9.552^a^	0.002
Haematuria (±−+)	70 of 307	58 of 268	12 of 39	1.611^a^	0.204
Clinical subtype					
Mild	6 (1.1)	6 (1.27)	0 (0)	0.898^a^	0.343
Ordinary	380 (69.98)	34 (72.94)	35 (50)	15.273^a^	<0.01
Severe	98 (18.05)	83 (17.55)	15 (21.43)	0.621^a^	0.431
Critically ill	59 (10.87)	39 (8.25)	20 (28.57)	26.012^a^	<0.01

Abbreviations: BNP, brain natriuretic peptide; CK‐MB, creatine kinase myocardial band; CRP, C‐reactive protein; eGFR, glomerular filtration rate; ESR, erythrocyte sedimentation rate; Fib, fibrinogen; PCT, procalcitonin; WBC, white blood cell.

The pulmonary lesions on the chest CT scan of these two cohorts differed from each other, in terms of position, size and shape (*P* < 0.05, Table 3). Specifically, patients with early renal impairment demonstrated pulmonary lesions of larger size, which were usually located at the central and peripheral lung at the same time. In addition, the pulmonary lesion in renal insufficient patients usually involved the whole lobe or segment of the lung.

**TABLE 3:** CT features of the lung in the two groups (Fisher's exact test)

Cohort	Total (*n*=)	Normal	Distribution		Position		Size		Shape	
			Unilateral	Bilateral	Periphery	Periphery and centre	≤3	>3	Patch	Lobes or segments
eGFR>60 ml/min 1.73m^2^	473	15	82 (17.3)	376 (79.5)	258 (54.6)	198 (41.9)	118 (25)	338 (71.5)	274 (57.9)	182 (38.5)
eGFR<60 ml/min 1.73m^2^	70	2	6 (8.6)	62 (88.6)	16 (22.9)	52 (74.3)	3 (4.3)	65 (92.9)	12 (17.1)	56 (80)
*P*			0.061		<0.01		<0.01		<0.01	

Abbreviations: CT, computed tomography; eGFR, glomerular filtration rate.

### 3.3 Identification of risk factors for in‐hospital death

Log rank test was performed to identify the parameters for prognostic significance. It was found that male gender, old age (>65 years old), specific complications (hypertension or diabetes mellitus), eGFR < 60 ml/min 1.73 m^2^ on admission, WBC > 9.5 × 10^9^/L, N% > 6.3 × 10^9^/L, L% < 1.1 × 10^9^/L, PLT < 125 × 10^9^/L, CRP > 10 mg/L, ALB < 35 g/L, PA < 200/L, BUN > 7.5 mmol/L and Cr > 73 μmol/L, as well as the chest CT that demonstrated that the pulmonary lesions involved the whole lobe or segment of the lung, and the clinical subtype of critically ill on admission were significantly associated with a higher mortality rate during hospitalization (*P* > 0.05, Table 4). Parameters with prognostic significance were further applied to the multivariate Cox regression analysis to determine the independent prognostic impact. As shown in Table 5, age >65 years old, the concurrence of diabetes mellitus, eGFR < 60 ml/min 1.73m^2^, WBC > 9.5 × 10^9^/L, ALB < 35 g/L or Cr > 73 μmol/L on admission were independently associated with the higher incidence of in‐hospital death.

**TABLE 4:** Cumulative incidence for in‐hospital death of patients with coronavirus disease 2019 (log‐rank test)

	Male	Age > 65 years old	Coexisting diseases				WBC (>9.5 × 10^9^/L)	*N* < 6.3 × 10^9^/L	*L* < 1.1 × 10^9^/L	PLT (<125 × 10^9^/L)	CRP (>10 mg/L)	Albumin (<35 g/L)
			Hypertension	Coronary heart disease	Diabetes	Renal diseases						
*χ* ^2^	10.474	13.157	9.589	0.017	18.275	2.712	62.933	4.883	16.584	6.395	17.623	23.542
*P*	0.001	<0.01	0.002	0.897	<0.01	0.1	<0.01	0.027	<0.01	0.011	<0.01	<0.01

Prealbumin (<200 g/L)	Urea nitrogen (>7.5 mmol/L)	Creatinine (>73 μmol/L)	GFR(<60)	Position (surrounding and centre)	Size (>3 cm)	Shape (lobes or segments)	Critically ill
8.045	56.802	7.888	36.967	1.019	3.674	8.903	286.322
<0.01	<0.01	0.005	<0.01	0.313	0.055	0.003	<0.01

Abbreviations: CRP, C‐reactive protein; GFR, glomerular filtration rate; PLT, significantly lower blood platelet; WBC, white blood cell.

**TABLE 5:** Multivariate cox regression analysis of in‐hospital death of patients with COVID‐19

	*B*	SE	Wald	Sig.	HRs	95.0% CI	
Male	−0.818	0.792	1.066	0.302	0.442	0.094	2.084
Age > 65 years old	1.484	0.585	6.445	0.011	4.412	1.403	13.877
Hypertension	0.289	0.712	0.165	0.684	1.336	0.331	5.392
Diabetes	−1.341	0.617	4.730	0.030	0.262	0.078	0.876
WBC > 9.5 × 10^9^/L	1.595	0.681	5.492	0.019	4.930	1.298	18.723
*N* > 6.3 × 10^9^/L	0.790	78.395	0.000	0.992	2.204	0.000	1.184E+067
*L* < 1.1 × 10^9^/L	−0.042	1.281	0.001	0.974	0.959	0.078	11.819
PLT < 125 × 10^9^/L	1.084	0.648	2.801	0.094	2.957	0.831	10.530
CRP > 10 mg/L	1.378	2.073	0.442	0.506	3.965	0.068	230.622
Albumin < 35 g/L	0.431	0.560	3.592	0.042	1.539	0.513	4.614
Urea nitrogen > 7.5 mmol/L	−1.063	0.632	2.824	0.093	0.345	0.100	1.193
Creatinine > 73 μmol/L	3.274	0.819	13.260	0.000	26.405	4.534	133.773
eGFR < 60 ml/min 1.73 m^2^	1.507	0.710	4.304	0.032	4.511	1.122	18.138
Shape (lobes or segments)	−0.534	0.671	0.634	0.426	0.586	0.157	2.183
Critically ill	−12.552	41.381	0.092	0.762	0.000	0.000	5.922E+29

Abbreviations: CRP, C‐reactive protein; eGFR, glomerular filtration rate; PLT, significantly lower blood platelet; WBC, white blood cell.

## 4 DISCUSSION

Through utilizing the available abundant clinical data on COVID‐19 in our hospital, the present retrospective study was conducted to unravel the clinical significance of renal impairment, which occurs at the early stage of COVID‐19 infection. It was found that early renal impairment in COVID‐19 patients was associated with more severe disease and worse clinical outcome. Early renal impairment is an independent risk factor for fatality in COVID‐19.

In a univariate survival analysis, it was found that male gender, age over 65 years old, complications such as hypertension or diabetes, elevation in WBC count or neutrophil count, decrease in lymphocyte count, thrombocytopenia and increase in CRP level may lead to increased risk of mortality. These present findings are consistent with the findings of the study conducted by Terpos et al.,^13^ which reported that COVID‐19 also has a significant impact on the haematopoietic system and coagulation function. In addition, both the univariate and multifactorial analyses revealed that early hypoproteinemia and renal dysfunction were independent risk factors of increased mortality.

Accumulated evidences have proven that renal impairment commonly occurs during coronavirus infection and conveys a non‐negligible clinical significance. Studies on previously identified coronaviruses, such as SARS and MERS, have revealed that AKI can occur in 5–15% of patients, and among these patients, the mortality rate can reach up to 60–90%.^14^, ^15^ A recent study that involved 193 cases of COVID‐19 reported the high prevalence of renal impairment among COVID‐19 patients, which specifically manifested as proteinuria (59%), haematuria (44%), elevated BUN (14%) and elevated creatine (10%). Furthermore, it was also reported that this may develop into AKI after prolonged hospitalization.^16^ Another recently published multicentre study that involved 1099 hospitalized patients with COVID‐19 revealed that the incidence of creatine ≥133 μmol/L was approximately 1.6%.^6^ The occurrence of renal impairment in COVID‐19 can be explained by the following mechanism: First, symptoms such as fever, loss of appetite and diarrhoea, which are caused by COVID‐19, can result in insufficient renal perfusion and subsequently lead to renal damage. Second, SARS‐CoV‐2 targets cells through the angiotensin‐converting enzyme 2 (ACE2) receptor, which is highly expressed in the lungs and even more abundantly expressed in the kidneys.^5^ SARS‐CoV‐2 can directly target the kidneys in an ACE2‐dependent pathway, as evidenced by the autopsy finding of glomerular and renal tubule swelling, cast in renal tubule, hyaline thrombi small vessels within the kidney and the detection of SARS‐CoV‐2 in urine.^17^ Third, the systemic inflammatory response and local sedimentation of the immune complex within the kidney can also result in renal damage.^18^

In the present study, the incidence of early renal impairment (eGFR <60 ml/min 1.73 m^2^ on admission) was 12.89%, and merely four cases (14.3%) were complicated with chronic renal diseases. It is noteworthy that 32.14% of patients with early renal impairment were critically ill, whereas merely 9.71% of patients with normal renal impairment developed severe COVID‐19. In addition, patients with impaired renal function had a significantly higher WBC count and CRP level, indicating that renal damage may be associated with the overwhelming inflammatory response caused by COVID‐19. Importantly, early renal impairment was significantly associated with prolonged hospitalization and higher mortality rate but was independent of all the other risk factors in predicting COVID‐19‐associated fatality, as shown by the multivariate Cox regression analysis. Similarly, a clinical trial on SRAS in 2003 revealed that concurrent AKI was associated with extremely high mortality, which can reach up to 91.7%, although the occurrence of AKI was not common in SARS patients. All these findings indicate that the concurrence of renal impairment in coronavirus diseases convey an unfavourable impact on clinical outcome. The kidney–lung crosstalk theory has been proposed to explain the interaction between kidney and lung damage and the consequent bidirectional deterioration.^19^, ^20^ Specifically, pulmonary injury and the clinical interventions applied in response to pulmonary function deterioration can jeopardize the homeostasis of kidney function.^20^ At the same time, acute kidney injury would alter the homeostasis of fluid balance, acid–base balance and vascular tone, resulting in the exacerbation of lung damage.^19^ Furthermore, kidney damage can also trigger a cascade of extra‐renal inflammatory responses, which further enhances the systemic inflammation derived from pneumonia.^19^, ^21^ The vicious cycle derived from the concurrence of lung and kidney damage can lead to irreversible exacerbation, multiple organ damage and even death.

The decline in prealbumin and albumin usually indicates the existence of malnutrition. As shown in the present results, patients with renal impairment had significantly lower prealbumin and albumin, when compared with the normal group. The decline in albumin and renal function insufficiency was found to be independently associated with higher risk of mortality for COVID‐19. Krieger reported that fatal malnutrition can result in the decline in liver weight and heart weight by 30%, as well as the significant decrease in renal perfusion and GFR.^22^ Other studies have also reported the decline in the metabolism of cytokine‐like interleukin‐6 in patients with hypoproteinemia, which further resulted in immune suppression.^23^, ^24^ The concurrent malnutrition can prolong the recovery period and increase the risk of infection.^25^ Nutrition support has been well‐accepted as one of the most important parts in the treatment and management of COVID‐19.^26^, ^27^

Several limitations in the present study need to be objectively addressed. First, as a single‐centre retrospective study, the sample size was moderate, and selection bias could not be avoided. Second, a small subset of patients received some anti‐virus medications for a short period of time before admission to the hospital. The confounding effect of these medications was neglected in the present study during the statistical analysis. Lastly, the study only focused on renal function on admission and did not further follow up the changes during the hospitalization. The changes on renal function along with disease progression and treatment and the correlation with clinical outcome need to be verified through further studies.

In conclusion, it was found that early renal impairment in COVID‐19 patients is associated with more severe disease and higher risk of in‐hospital death in COVID‐19. Risk stratification according to renal functional status and enhanced intervention strategies for high risk patients can better improve the prognosis of COVID‐19.

## CONFLICT OF INTEREST

The authors declare that they have no conflict of interest.

## ETHICS APPROVAL AND CONSENT TO PARTICIPATE

The present study was approved by the ethics committee of our hospital (Ethics committee approval number: XGLY2020‐04‐11). All procedures performed in studies involving human participants were in accordance with the ethical standards of the institutional and/or national research committee and with the 1964 Helsinki declaration and its later amendments or comparable ethical standards.

## CONSENT FOR PUBLICATION

All data published here are under the consent for publication. A written informed consent was obtained from all individual participants included in the study.

## AUTHOR CONTRIBUTIONS

Pei‐ling Bao, Ke‐lan Deng and An‐long Yuan performed most of the investigation and data analysis and wrote the manuscript. Yi‐min Yan performed Statistical analysis. Ai‐qiao Feng and Tao Li contributed to interpretation of the data and analyses. Xiao‐an Liu directed manuscript writing. All of the authors have read and approved the manuscript.

## Data Availability

The datasets generated and analysed during the present study are available from the corresponding author on reasonable request.
